# Peri-Vesical Fat Interposition Flap Reinforcement in High Vesico-Vaginal Fistulas

**DOI:** 10.4103/2006-8808.73617

**Published:** 2010

**Authors:** R. B. Singh, S. Dalal, S. Nanda

**Affiliations:** *Department of Burns and Plastic Surgery and Hypospadias and VVFs Clinic, Postgraduate Institute of Medical Sciences, Rohtak - 124 001, Haryana, India*; 1 *Department of General Surgery, Postgraduate Institute of Medical Sciences, Rohtak - 124 001, Haryana, India*; 2 *Department of Obstetrics and Gynecology, Postgraduate Institute of Medical Sciences, Rohtak - 124 001, Haryana, India*

**Keywords:** Interposition flap, peri-vesical fat flap, vesico-vaginal fistulas

## Abstract

**Background and Aim:**

The urinary bladder becomes small, contracted and is associated with excess pelvic fat in long standing cases of vesico-vaginal fistulas (VVFs). The aim of this new technique was to use this excess pelvic fat for harvesting an interposition flap.

**Materials and Methods:**

An interposition flap of peri-vesical fat was raised from the anterior, superior and posterior surfaces of the urinary bladder and was interposed between the right angle closed vaginal vault and the urinary bladder to strengthen the repair. This technique was used in two patients of VVFs.

**Results:**

Both the patients had successful outcome and were able to retain sufficient quantity of urine at 3 months follow-up.

**Conclusions:**

Peri-vesical fat flap proved an effective interposition flap in the repairs of VVFs in selected cases.

## INTRODUCTION

The formation of vesico-vaginal fistulas (VVFs) is the worst complication that can still be seen in developing and developed countries. Lack of proper obstetric care and prolonged obstructed labor forms the main cause in developing countries, while radical hysterectomy and radiotherapy for malignancy is the leading cause in developed countries.[1] Various methods of VVF repair have been described in literature which include open transabdominal, transvaginal and laparoscopic approaches, depending on the characteristics of the fistula.[2]

Large number of reinforcement flaps (interposition flaps, on-lay flaps) have also been described in literature for strengthening the repairs of these VVFs.[3–5] A new technique for designing and harvesting an interposition flap from the pelvic fat surrounding the urinary bladder is described here.

## MATERIALS AND METHODS

**Figure 1:**
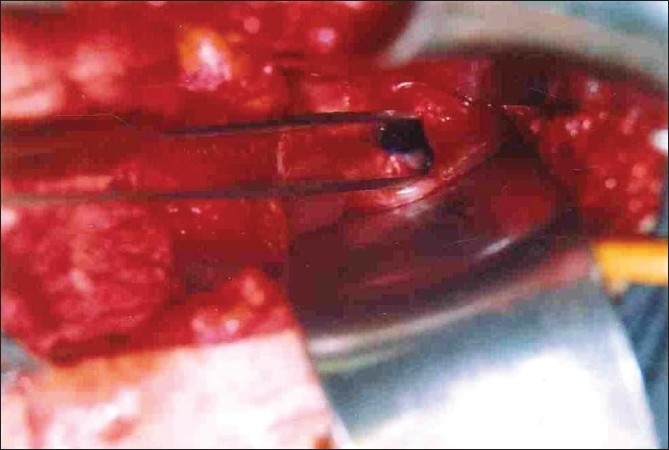
2 × 3 cm sized VVF

Two women aged 30 and 35 years had developed 2 × 2 cm and 2 × 3 cm sized high VVFs, respectively, as a result of total abdominal hysterectomy carried out for dysfunctional uterine bleeding [Figure 1]. Both the patients were subjected to detailed history and necessary clinical examination including bimanual, per speculum examination and cystoscopy. The mandatory investigations done were blood urea, serum creatinine, USG of pelvic organs and culture and sensitivity of urine and vaginal swab. Antibiotics were started 1 day before surgery and were continued till all the tubings were removed. For surgery, both the cases were positioned with head side low, i.e., Trendlenberg’s position, for transperitoneal-transvesical approach. Both the cases could be done under spinal anesthesia. On exploration, fistula margins were thick, soft and supple in both the cases.

**Figure 2:**
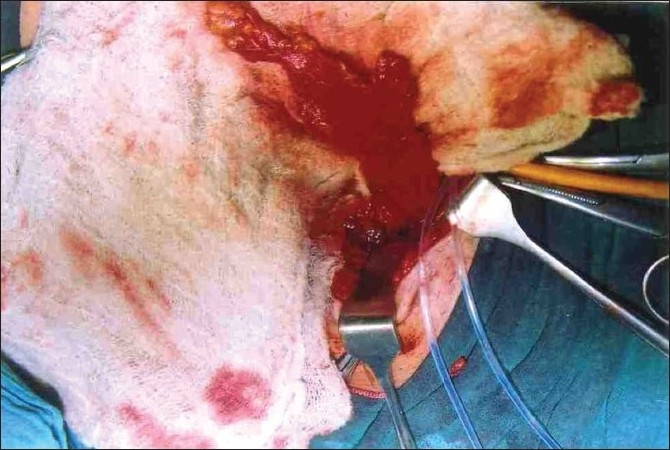
Peri-vesical fat flap harvested

**Figure 3:**
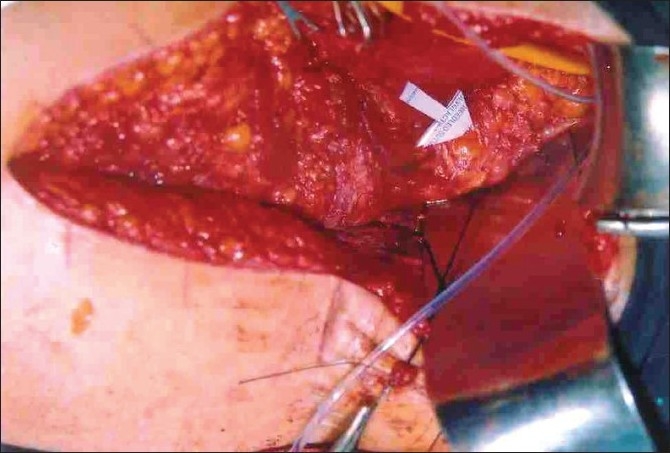
Peri-vesical fat flap reinforcing the closed vaginal vault

During transabdominal exploration, the urinary bladder could not be visualized due to the presence of excessive amount of the fat in the pelvis. We decided to utilize this fat to design peri-vesical fat flap. Approximately 5 cm wide and 1 cm thick flap of the fat was raised from the anterior, dome and the posterior aspects of the wall of the deeply hidden urinary bladder [Figure 2]. After wide separation, the vaginal vault and the urinary bladder were closed separately at right angles to each other in two layers, inner continuous and outer interrupted using 2-0 vicryl on round body needle. The peri-vesical fat flap was interposed between them to act as a biological as well as mechanical barrier against re-fistulizations [Figure 3]. Intraperitoneal pelvic drain, suprapublic catheter and Foley’s catheter were inserted and adequately fixed to prevent their kinking and mechanical blockage. A vigilant postoperative care was ensured to both the patients to maintain high urine output to prevent intraluminal blockage of catheters. They were put on semisolid diet and stool softeners to prevent straining during stools.

## RESULTS

The postoperative course was smooth in both the patients, with no major complication. They were discharged from hospital after 2 weeks of repair in a healthy condition when all their tubings and stitches were removed. Patients were evaluated two weekly for the initial 3 months and both the patients were asymptomatic and continent at 3 months of follow-up. They had 200 ml of the urine holding capacity of their urinary bladder. Three monthly follow-up was advised thereafter. Since both the patients were sexually active, abstinence from sexual intercourse was advised for a minimum of 3 months.

## DISCUSSION

Despite improvements in surgical techniques and obstetrical care, the VVFs still occur and keep on testing the surgical skills of the surgeons and gynecologists. Various methods of fistula repair have been described in the literature, which include transabdominal, transvaginal and laparoscopic methods, depending on the characteristics of the fistula.[2] In spite of the management being better defined in the last decade, the surgical approach has always been an issue of contention. The fundamental principles of repair, i.e., adequate exposure, tension-free approximation of fistula edges, multilayered closure of bladder and vagina at right angles to each other, good hemostasis, etc., can be achieved through both vaginal and abdominal routes. However, when the fistula is complex, vaginal exposure of the fistula is suboptimal which may compromise the repair or endanger the ureters. In these cases, a transabdominal approach should be preferred.[2] Both of our patients were having complex fistulas because of their high locations and so were operated by abdominal route.

Recurrence is the main postoperative complication associated with all kinds of VVF repairs, and to prevent this, all repairs are mandatory to be strengthened by routine use of reinforcement flaps. Omental flap in transperitoneal approach and Martius flap in transvaginal approach are two such reinforcement flaps which are most versatile and vascular and can be harvested with least discomfort and without producing any functional and cosmetic deformities.[4] Besides these two, a number of other reinforcement flaps have been described in literature to prevent fistulizations following repairs of VVFs.[3–6] The authors themselves have been using both these flaps routinely in most patients undergoing repairs of VVFs with successful outcomes. Few such flaps have originally been devised and used by authors and they were not mentioned in the literature before. They include interposition flaps using tissues from broad ligaments, fallopian tubes, re-use of Martius flap, etc.[3,5,6]

The other problem in chronic VVF cases is reduced capacity of urinary bladder, which is associated with small, contracted bladder with excess pelvic fat surrounding the bladder. We have successfully used this pelvic fat as an interposition flap in two patients to further strengthen the VVF repairs. The present technique makes beneficial and healthy use of pelvic fat and prevents additional donor site morbidity and disfigurements. The only disadvantage with this technique is that it is possible only in a limited number of patients having a thick layer of pelvic fat hiding the urinary bladder.

## CONCLUSIONS

Peri-vesical fat flap can prove as an effective interposition flap and the authors recommend the use of this technique to strengthen the VVF repair in selected cases associated with excess pelvic fat.
